# Role of family support and self-care practices in blood pressure control in individuals with hypertension: results from a cross-sectional study in Kollam District, Kerala

**DOI:** 10.12688/wellcomeopenres.16146.1

**Published:** 2020-07-28

**Authors:** Susanna Chacko, Panniyammakal Jeemon

**Affiliations:** 1 Achutha Menon Centre for Health Sciences Studies, Sree Chitra Thirunal Institute for Medical Sciences and Technology, Trivandrum, Kerala, 695011, India

**Keywords:** Hypertension, blood pressure control, self-care, family support, India

## Abstract

**Background**: Despite the availability of effective drugs, blood pressure (BP) control rate is sub-optimal in individuals with hypertension in low- and middle-income countries (LMICs). The role of self-care in the management of BP is less studied in LMIC settings.

**Methods**: We conducted a community-based, cross-sectional study in individuals with hypertension in Kollam district, Kerala. A multistage cluster sampling method was used for the selection of study participants. We measured self-care by using an adapted Hypertension Self-Care Activity Level Effects (H-SCALE) scale. Descriptive statistics were used to summarise the data and logistic regression analysis was conducted to identify factors associated with BP control.

**Results**: In total, 690 individuals with hypertension (women=60%) and a mean age of 57±8 years participated in the study. More than half (54%) of the participants were adherent to anti-hypertensive medications. However, the adherence rate was much lower for the dietary approach to stop hypertension (DASH) diet (12.8%), recommended level of physical activity (24%) and weight management (11.4%). Overall BP control was achieved in two of five individuals (38.4%, 95% CI: 34.7-42.0%). Among self-care activities, adherence to medications (AOR: 1.8, 95% CI: 1.3-2.5), DASH diet (AOR: 1.5, 95% CI: 1.0-2.4), and non-smoking status (AOR: 3.3, 95% CI: 1.7-6.4) were associated with control of BP. Additionally, good family support to self-care (AOR: 1.9, 95% CI: 1.1-3.1) was associated with better control of BP.

**Conclusion:** In individuals with hypertension, the BP control rate is achieved in two of five individuals. Adoption of self-care activities are sub-optimal. Both family support and adherence to self-care activities are associated with BP control.  Family based interventions to improve adherence to self-care activities could have a significant public health impact in achieving better population-level BP control rates in Kerala, India.

## Introduction

Globally, hypertension is a major cause of premature death and disability. Two of five adult individuals worldwide have raised blood pressure (BP) ^1^. Annually, more than 10 million deaths and 200 million disability adjusted life years (DALYs) are attributable to elevated BP ^2^. Hypertension is undoubtedly a major risk factor for stroke, heart failure, coronary heart disease and chronic kidney disease. More than half of the deaths due to cardiovascular disease (CVD) are linked to complications from hypertension ^3^. Hypertension is also a major public health problem in India, affecting almost one in three adult individuals ^4^. Additionally, high BP is attributable to more than 1.5 million deaths and 38 million DALYs in India ^5^.

Optimal BP control is essential to prevent the premature deaths and related disability attributable to hypertension. For example, a reduction of more than a third in the risk of stroke and a reduction of a quarter in the risk of myocardial infarction and heart failure are associated with optimal BP control in individuals with hypertension ^6^. However, BP control rates are abysmal in low- and middle-income countries (LMICs) even with the availability of effective drugs for management of hypertension ^7^. The disproportionately higher number of deaths from elevated BP in LMICs as compared to high-income regions is largely attributable to sub-optimal control of BP.

Hypertension is prevalent in two of five adult individuals in the state of Kerala, India ^8^. Despite the availability of a relatively strong public health system ^9^, BP control rate is poor in Kerala. For example, the BP control rate at the population level in adults over 18 years was less than 15% in a recent state-wide representative survey conducted in Kerala ^8^. In order to achieve optimal BP control, it is desirable to identify the major road blocks and target them with comprehensive health system approaches.

Comprehensive management of hypertension requires both pharmacological and lifestyle modification. Self-care has been recognised as an important determinant for achieving optimal BP control at the individual level. According to the World Health Organisation (WHO), self-care is defined as “the ability of individuals, families and communities to promote health, prevent disease, maintain health and to cope with illness and disability with or without the support of a healthcare provider” ^10^. Further, hypertension guidelines advise self-care activities such as self-monitoring of BP, reduction of dietary sodium intake, increase in physical activity, limiting alcohol intake, adoption of a dietary approach to stop hypertension (DASH) diet plan, weight management and abstinence from tobacco as lifestyle modification strategies to achieve optimal BP control ^11^. However, research in the areas of self-care practices and BP control are limited in LMICs. We aimed to assess hypertension self-care practices and its impact on BP control among adult individuals with hypertension in Kerala.

## Methods

### Ethical statement

The Institutional Ethics Committee of Sree Chitra Tirunal Institute for Medical Sciences and Technology, Trivandrum, Kerala reviewed and approved the conduct of the study in Kerala (SCT/IEC/1450/NOVEMBER-2019). Written informed consent was obtained from all the study participants before administering the interview schedule. The participants had the freedom to refuse participation at the outset or during any stage of the study. There was no anticipated risk for participants by their involvement in the study. None of the selected participants refused participation.

### Study design

We conducted a community based cross-sectional survey in Kollam District, Kerala, India.

### Study setting

The study was conducted in five of eleven randomly selected ‘ *block panchayats’* (panchayats are the lowest level of the three-tier local self-governance model in Kerala) of Kollam district, Kerala, India and the data collection took place between December 2019 to February 2020. The population size of Kollam district is 2.64 million, based on the 2011 census ^12^. 

### Study population

Individuals in the age group of 30–70 years, diagnosed with hypertension for at least six months and started on at least one antihypertensive drug as part of initial management were eligible to be part of the study. Individuals who could not communicate and were unable to provide informed consent were excluded from the study. A multistage cluster sampling method was used to identify eligible participants for the cross-sectional survey conducted in Kollam district, Kerala. The primary sampling unit was ‘ *block panchayats*’. Initially, five of eleven ‘ *block panchayats*’ were randomly selected from Kollam District, Kerala using computer-generated random numbers. In the second stage of sampling, two primary health centres (PHCs: caters to a population of approximately 25,000–40,000) were identified by simple random sampling procedure from each ‘ *block panchaya*t’. Additionally from each primary health centre, we randomly identified three sub-centres (sub-centres cater to a population of approximately 5,000 individuals) for the study using computer-generated random numbers. We obtained the listing of all individuals with hypertension in the selected sub-centres based on the population-based screening records held by the Accredited Social Health Activists (ASHA) in the respective areas. From the chosen sub-centres, we randomly selected 23 individuals with hypertension using computer-generated random numbers from the population-based listing of all individuals with diagnosed hypertension. Participants were approached in person at their residence by the researcher (SC) with the help of a local community health worker (ASHA). Participants were provided with an information sheet (see *Extended data* ^13^) in Malayalam language, which contains details about research aims, objectives, and information regarding study questionnaires.

### Sample size

A sample size of 690 individuals with hypertension yields more than 80% power to estimate reliably a range of prevalence of self-care activities and BP control rate from 10% to 50% ^8, 14^ with an absolute precision of 2.5% (assuming an alpha error of 0.05 and a design effect of 1.5).

### Study variables and data collection

The researcher (SC) collected the data in person by visting each study participant at their residence. We collected information regarding sociodemographic factors, health-seeking behaviour, details related to comorbidities, family support and self-care practices. Self-care activities were captured using the adapted version of Hypertension Self-care Activity Levels Effects (H-SCALE) developed by Jan Warren-Fellow and Seymour ^15^. The H-SCALE was modified to suit the cultural practices prevalent in Kerala. Questions related to intake of certain types of fruits and vegetables were modified and replaced with culturally relevant food items.A panel of experts with expertise in public health, epidemiology and clinical cardiology reviewed the face validity and content validity of the modified version of the questionnaire (see *Extended data* ^16^). The modified tool was translated into the local language (Malayalam, see *Extended data* ^17^), and piloted in five individuals with hypertension who were selected from Trivandrum district in Kerala. The piloting involved testing the wordings, possible responses, and clarity of instructions. The tool was finalised after an expert review of the responses received to each item of the scale in the piloting phase. Only minor changes to the wording of some of the items were introduced to improve clarity of the questions. We measured BP and heart rate of the study participants using a digital sphygmomanometer (OMRON HEM-7121). BP was measured after the individual was seated comfortably in an upright position with their arm rested and using an appropriate cuff-size. The BP was measured from the left arm as per the WHO guidelines. After obtaining the first reading, the cuff was deflated fully and the next measurement was conducted after three minutes. The measurement was repeated for the third time by following the same procedure. The mean of the consecutive three readings was obtained and recorded as the BP of the individual. 

### Definitions

*Medication adherence*: We used a three-item scale to measure how many days the person took the medication in a week at the recommended dosage and at the recommended time. The scores for each item were summed (range 0–21). A score of 21 was considered as good adherence.

*Adherence to the DASH diet*: We used an 11-item scale to assess the intake of healthy foods associated with the nutritional composition of the DASH diet. The scores for each item were summed. The possible range of scores for the DASH-Q scale was 0 to 77. A score of <32 was considered as low diet quality; a score between 33 and 51 was considered as medium quality, and scores of 52 or greater were considered as good adherence.

*Physical activity engagement*: A two-item scale measured the number of days of physical activity of at least 30 minutes for each participant. The scores on both items were summed (range 0-14). A score of eight or above was considered as good adherence to physical activity.

*Smoking*: The scores in a two-item scale were summed (range 0-14). Respondents who scored zero were considered as adherent to non-smoking.

*Weight management*: A 10-item scale measured weight management activities in the last month. The sum of the scores on all items ranged from 10-50. Individuals who scored above 40 were considered as adherent to weight management practices.

*Alcohol use*: Individuals who did not drink alcohol at all were considered as alcohol abstinent.

*Family support*: A 16- item scale measured the influence of family members on diet and other health behaviour. The average score on all items ranged from 0.94–2.56. We categorised family support into minimal, mild, moderate and strong based on quartiles of the observed score. A score of <1.37, 1.38–1.56, 1.57–1.68, and 1.69 or greater were considered as minimal, mild, moderate and strong support, respectively.

*BP control*: BP control was defined as a mean BP ≤140/90 mmHg in all individuals based on the average of three readings.

### Data analysis

We performed all data analysis in SPSS Version 25. Categorical variables were presented as frequencies and percentages. Continuous variables were presented as means with standard deviations. We performed multivariate logistic regression analysis to identify independent factors associated with BP control. All variables found to be statistically associated with BP control in the bivariate analyses at a higher p-value threshold of 0.1 were considered in the multivariate model.

## Results

### General characteristics of the study population

In total, 690 individuals (response rate = 100%) participated in the study. The age of participants ranged from 31 to 70 years with a mean age of 57±8 years ( Table 1). More than half of the participants (59.7%) were women. More than one-quarter (29%) of participants had primary or less than primary level of education. One in 10 women (10.7%) were living alone, while this was true for 3% of men. Below poverty level (BPL) ration cards were held by 44% of participants. Individual-level data for each participant are available as *Underlying data* ^18^.

**Table 1.** General characteristics of the study population.

Characteristics	Men N=278	Women N=412	p-value
**Age, mean (SD)**	57.40 (±8.91)	56.93 (8.73)	0.489
**Age group, n (%)**			0.520
31 - 40	16 (5.8)	23 (5.6)	
41-50	41 (14.7)	78 (18.9)	
51-60	109 (39.2)	147 (35.7)	
61-70	112 (40.3)	164 (39.8)	
**Marital status, n (%)**			0.0001
Married	249 (89.6)	267 (64.8)	
Single /divorced	29 (10.4)	145 (35.2)	
**Education, n (%)**			0.360
Primary	74 (26.6)	127 (30.8)	
Secondary	146 (52.5)	213 (51.7)	
Higher secondary	58 (20.9)	72 (17.5)	
**Occupation, n (%)**			0.001
Self/formal employment	89 (32.0)	50 (12.1)	
Unemployed	37 (13.3)	27 (6.6)	
Retired	45 (16.2)	32 (7.8)	
Homemakers	0	262 (63.6)	
Daily wages	107 (38.5)	41 (10.0)	
**Living alone, n (%)**			0.001
Yes	9 (3.2)	44 (10.7)	
No	269 (96.8)	368 (89.3)	
**Income categories, n (%)**			0.262
Below poverty line	114 (41.0)	188 (45.6)	
Above poverty line	164 (59.0)	224 (54.4)	

### BP related characteristics of the study population

The mean age of diagnosis of hypertension was 50±9 years ( Table 2). Nearly three of five participants (59%) measured their BP at least once in a month. However, three-quarters (74.8%) of individuals were not aware of their last BP values. More than half (54%) were seeking treatment from public facilities. Similarly, more than half of the study participants (52.5%) visited a physician or health worker at least on a monthly basis. Only 10.8% and 7.3% of men and women were monitoring BP at home, respectively.

**Table 2.** Blood pressure, health seeking pattern and adherence to self-care components.

Characteristics	Men	Women	p-value
**Age at diagnosis, mean (SD)**	50.50 (9.5)	50.62 (9.3)	0.871
**Duration of hypertension, mean (SD)**	6.90 (6.6)	6.31 (6.3)	0.237
**Systolic BP (mmHg), mean (SD)**	142.55 (20.7)	144.59 (19.4)	0.186
**Diastolic BP (mmHg), mean (SD)**	87.55 (11.5)	86.90 (11.3)	0.465
**Health care facility, n (%)**			0.840
Public	150 (54.0)	228 (55.3)	
Private	84 (30.2)	116 (28.2)	
Both	44 (15.8)	68 (16.5)	
**Visit to a physician/health worker, n (%)**			0.433
Weekly/monthly	146 (52.5)	198 (48.1)	
Twice/once in a year	53 (19.1)	79 (19.2)	
Rarely	79 (28.4)	135 (32.8)	
**Home BP monitoring, n (%)**			0.142
Yes	30 (10.8)	30 (7.3)	
No	248 (89.2)	382 (92.7)	
**Awareness of last BP value, n (%)**			0.432
Yes	75 (27.0)	99 (24.0)	
No	203 (73.0)	313 (76.0)	
**Frequency of BP monitoring, n (%)**			0.220
Monthly	175 (62.9)	232 (56.3)	
Twice/once in a year	37 (13.3)	65 (15.7)	
When symptoms occur/rarely	66 (23.7)	115 (28.0)	
**Perceived BP control status, n (%)**			0.143
Yes	115 (41.4)	195 (47.3)	
No	163 (58.6)	217 (52.7)	
**Family support in self-care, n (%)**			0.065
Minimal	66 (23.7)	118 (28.6)	
Mild	82 (29.5)	138 (33.5)	
Moderate	55 (19.8)	78 (18.9)	
Strong	75 (27.0)	78 (18.9)	
**Self-care practices, n (%)**			
Medication adherence	168 (60.4)	210 (51.0)	0.018
DASH diet adherence	40 (14.4)	48 (11.7)	0.347
Physical activity adherence	100 (36.0)	65 (15.8)	<0.001
Weight management adherence	44 (15.8)	37 (9.0)	0.009
Alcohol abstinence	241 (86.7)	412 (100.0)	<0.001
Non-smoking	215 (77.3)	412 (100.0)	<0.001

BP, blood pressure; SD, standard deviation; DASH, dietary approach to stop hypertension.

Hypertension in isolation was present in 30% of the study population. Diabetes (39.1%) and dyslipidemia (32.2%) were the major comorbid conditions. The proportion of study participants with hypertension and one or two or more co-morbidities were 39% and 31.2%, respectively.

### Self-care practices

The overall prevalence of medication adherence among study participants was 54.8% ( Table 2). The medication adherence was higher in men (60.4%) as compared to women (51%). Adherence to the DASH diet was very poor in both men (14.4%) and women (11.7%). Overall, 24% of participants were engaged in the recommended level of physical activity. Adherence to the recommended level of physical activity was very low in women (15.8%) as compared to men (36%). Nearly one-third (32.7%) of men were smokers. Alcohol use was prevalent in 13.3% of men. Overall, 11.4% of the individuals were adherent to weight management practices ( Table 2).

### Family support in self-care

More than half (53.2 %) of men and 62.1% of women reported minimal to mild family support to self-care activities. Similarly, 47.6% and 37.7% of men and women reported moderate to strong family support to self-care activities related to hypertension management, respectively ( Table 2). 

### Factors associated with BP control

**Figure 1.**
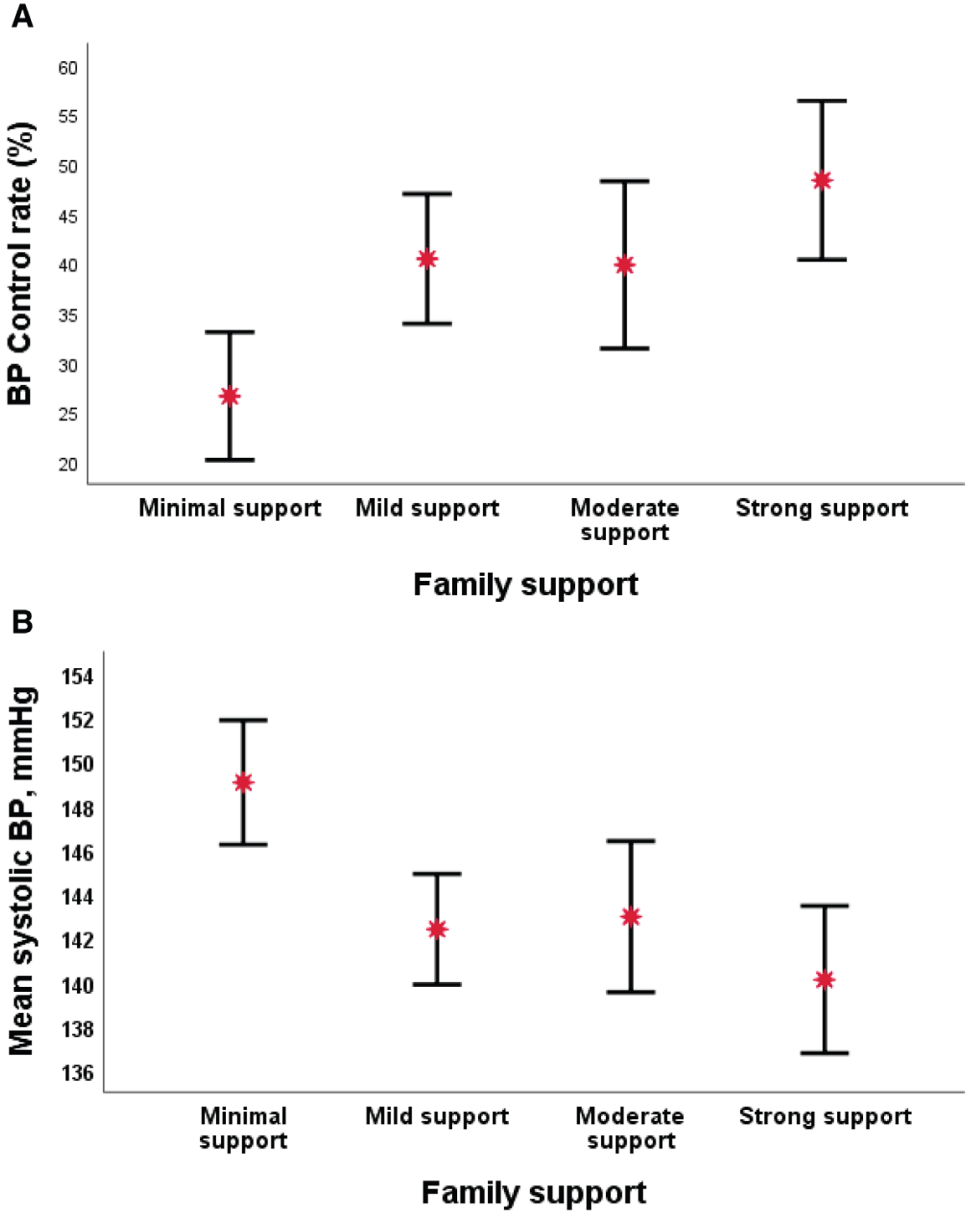
Panel **A**) Family support and blood pressure (BP) control rate. Panel **B**) Family support and mean systolic BP.

The proportion of individuals with controlled BP increased with the improvement in family support from minimal (26.6%) to strong support (48.8%). Similarly, the mean systolic BP was lowest in individuals with strong family support to self-care activities (140.10±2.82 mmHg) as compared to individuals with minimal family support (149.05±3.35mmHg) ( Figure 1).

Age group, gender, educational level or occupation did not show any association with control of BP in the bivariate analysis ( Table 3). Similarly, duration of hypertension, home BP monitoring and frequency of BP monitoring were not associated with BP control. Only a quarter of patients living alone (24.5%) achieved BP control as compared to 40% in patients living with other family members (p = 0.04). Individuals seeking care from both private and public facilities were less likely to achieve BP control as compared to individuals seeking care from either private or public facilities. BP control rate improved with family support, adherence to medications and DASH diet. BP control rate was also better in those who were adherent to non-smoking practices as compared to smokers ( Table 3).

**Table 3.** Factors associated with blood pressure control.

Variable	Controlled n = 265	Uncontrolled n = 425	p-value
**Age group, n (%)**			0.429
31 - 40	18 (46.2)	21 (53.8)	
41 - 50	42 (35.3)	77 (64.7)	
51 - 60	105 (41.0)	151 (59.0)	
61 - 70	100 (36.2)	176 (63.8)	
**Gender, n (%)**			0.283
Men	114 (41.0)	164 (59.0)	
Women	151 (36.7)	261 (63.3)	
**Education level, n (%)**			0.730
Up to primary	78 (38.8)	123 (61.2)	
Secondary	141 (39.3)	218 (60.7)	
Higher secondary and above	46 (35.4)	84 (64.6)	
**Occupation, n (%)**			0.962
Formal/self-employment	56 (40.3)	83 (59.7)	
Unemployed	25 (39.1)	39 (60.9)	
Retired	27 (35.1)	50 (64.9)	
Homemaker	101 (38.5)	161 (61.5)	
Daily wages	56 (37.8)	92 (62.2)	
**Living alone, n (%)**			0.044
Yes	13 (24.5)	40 (75.5)	
No	252 (39.6)	385 (60.4)	
**Health care facility, n (%)**			0.006
Public	154 (40.7)	224 (59.3)	
Private	83 (41.5)	117 (58.5)	
both	28 (25.0)	84 (75.0)	
**Duration of hypertension, n (%)**			0.245
<5 years	136 (37.9)	223 (62.1)	
5-10 years	74 (35.6)	134 (64.4)	
<10 years	55 (44.7)	68 (55.3)	
**Home BP monitoring, n (%)**			0.337
Yes	27 (45.0)	33 (55.0)	
No	238 (37.8)	392 (62.2)	
**Frequency of BP monitoring, n (%)**			0.922
Monthly	155 (38.1)	252 (61.9)	
Twice/once in a year	41 (40.2)	61 (59.8)	
When symptoms occur/rarely	69 (38.1)	112 (61.9)	
**Family support, n (%)**			0.001
Minimal	49 (26.6)	135 (73.4)	
Mild	89 (40.5)	131 (59.5)	
Moderate	53 (39.8)	80 (60.2)	
Strong	74 (48.4)	79 (51.6)	
**Medication adherence, n (%)**			<0.001
Adherent	169 (44.7)	209 (55.3)	
Non-adherent	96 (30.8)	216 (69.2)	
**DASH diet adherence, n (%)**			0.071
Adherent	42 (47.7)	46 (52.3)	
Non-adherent	223 (37.0)	379 (63.0)	
**Physical activity adherence, n (%)**			0.261
Adherent	70 (42.4)	95 (57.6)	
Non-adherent	195 (37.1)	330 (62.9)	
**Weight management adherence, n (%)**			0.924
Adherent	32 (39.5)	49 (60.5)	
Non-adherent	233 (38.3)	376 (61.7)	
**Alcohol abstinence, n (%)**			0.552
Adherent	253 (38.7)	400 (61.3)	
Non-adherent	12 (32.4)	25 (67.6)	
**Non-smoking adherence, n (%)**			0.001
Adherent	253 (40.4)	374 (59.6)	
Non-adherent	12 (19.0)	51 (81.0)	

BP, blood pressure; DASH, dietary approach to stop hypertension.

In the multivariate model ( Table 4), participants who were adherent to medication were approximately 1.5 times more likely to have controlled BP than non-adherent individuals (P = 0.009, OR = 1.5, 95% CI = 1.1-2.2). Individuals who were adherent to the DASH diet were 1.6 times more likely to have controlled BP as compared to the non-adherent group (P = 0.043, OR = 1.6, 95% CI = 1.0-2.6). Similarly, adherence to non-smoking improved the odds of BP control by almost three times (P = 0.001, OR = 3.1, 95% CI = 1.6-6.3).

**Table 4.** Strength of association of self-care and control of blood pressure.

Variable	Unadjusted OR (95% CI)	p-value	Adjusted OR (95% CI)	p-value
**Living alone** Yes No	Reference 2.0 (1.1-3.9)	0.034	Reference 1.7 (0.8-3.5)	0.116
**Family support** Minimal Mild Moderate Strong	Reference 1.9 (1.2-2.9) 1.8 (1.1-2.9) 2.6 (1.6-4.0)	0.004 0.013 <0.001	Reference 1.4 (0.9-2.2) 1.3 (0.8-2.8) 1.9 (1.1-3.1)	0.124 0.218 0.013
**Health care facility** Public Private Both	Reference 1.0 (0.7-1.5) 0.5 (0.3-0.8)	0.860 0.003	Reference 0.9 (0.6-1.3) 0.5 (0.3-0.8)	0.648 0.005
**Adherence to home BP monitoring** No Yes	Reference 1.3 (0.8-2.3)	0.273	*	
**Medication adherence** Adherent Non-adherent	1.8 (1.3-2.5) Reference	<0.001	1.5 (1.1-2.2) Reference	0.009
**DASH diet adherence** Adherent Non-adherent	1.5 (1.0-2.4) Reference	0.056	1.6 (1.0-2.6) Reference	0.043
**Physical activity adherence** Adherent Non-adherent	1.2 (0.9-1.8) Reference	0.224	*	
**Weight management adherence** Adherent Non-adherent	1.1 (0.7-1.7) Reference	0.828	*	
**Non-smoking adherence** Adherent Non-adherent	3.3 (1.7-6.4) Reference	<0.001	3.1 (1.6-6.3) Reference	0.001
**Alcohol abstinence** Adherent Non-adherent	1.3 (0.7-2.7) Reference	0.444	*	

BP, blood pressure; OR, odds ratio; CI, confidence interval; DASH, dietary approach to stop hypertension. * Not included in multivariate analysis

## Discussion

BP control is an important treatment goal for prevention of cardiovascular disease and related complications in individuals with hypertension. Self-care practices are sub-optimal and they are strongly associated with BP control in individuals with hypertension in Kerala. Additionally, family support enhances adherence to self-care practices related to BP management.

The overall BP control rate in our study population is higher than the data reported in previous studies from Kerala. For example, a large cross-sectional survey conducted in Kerala observed that BP control is only achieved in less than 15% of individuals with hypertension ^8^. As a standard practice in community-based surveys on hypertension prevalence, the above cited study included all individuals with elevated BP above the hypertension threshold of 140/90 mmHg in the ‘uncontrolled BP’ category. Hence, even the individuals with a first time diagnosis of hypertension based on a single visit BP measurement are labelled as ‘uncontrolled hypertension’. However, we have included those who were diagnosed with hypertension for at least six months, started on at least one antihypertensive drug as part of initial management, and had a BP above the hypertension threshold in the ‘uncontrolled BP’ category in our study. Therefore, the control rate observed in our study is among individuals who were formally diagnosed and treated for hypertension. Unlike other facility-based surveys, our results are based on a representative community-based sample of individuals with diagnosed hypertension.

Self-care practice was less than optimal in our study population. Despite the prescription of treatment and the follow-up by ASHA workers, only 55% were adherent to the medications. Additionally, adherence to DASH diet components were only observed in less than 15% of the study population. Our study demonstrates that self-care practices are very important in achieving optimal control of BP. We show that those who were adherent to drugs, the DASH diet and non-smoking achieved better BP control than the non-adherent group. Consistent findings on adherence to medication and BP control are reported in other studies ^19– 21^.

BP control in individuals with hypertension often requires adherence to self-care activities beyond medications. However, self-care activities related to diet and smoking are often ignored. Clinical trial evidence suggests that a low sodium DASH diet improves the control of BP and reduces cardiovascular risk in individuals with hypertension ^11^. In the PREMIER trial, the DASH diet and other lifestyle practices together reduced BP and cardiovascular events ^22^. Despite the known benefits of a low sodium DASH diet, adherence to DASH diet components were abysmally poor in our study. Community-based strategies to improve adherence to DASH diet components ^23^ and policy initiatives promoting low sodium salt ^24^ may improve the BP control rate at the population level. Prescribing mandatory targets for the food industry, front of pack labelling, food procurement policies and taxation are some additional policy initiatives to reduce population level salt consumption ^25^.

We show that smoking status is independently associated with BP control. Consistent findings are reported in the national health survey in England ^26^. Available evidence also shows that nicotine in cigarettes acts as an adrenergic agonist and mediates release of catecholamines, which affects BP levels and heart rate ^27^. Alcohol abstinence is also an important self-care practice that plays a significant role in the control of BP. In our study, alcohol use was not associated with BP control. However, the amount of alcohol consumption should be taken into consideration while exploring the association with BP control. In the INTERSALT study, a significant reduction in BP was observed in people who had limited their alcohol consumption ^28^. The potentially causal relationship between alcohol abstinence and BP reduction in the INTERSALT study provides further evidence to recommend alcohol reduction to control BP in hypertensive individuals.

Family support of self-care activities is a key factor associated with BP control in our study. Better family support acts through improvements in the adherence to self-care activities and thereby improves BP control. The positive association between BP control and perceived family support emphasizes the need for health care providers to assess the available family support when managing individuals with hypertension. Further, it calls for innovative family-based models such as the Programme of Lifestyle Intervention in Families (PROLIFIC study) in managing BP ^29^. The PROLIFIC model vouches for a family centred strategy for lifestyle changes and self-care for cardiovascular risk reduction. In a family centred approach, the proposed lifestyle changes and self-care strategies are more achievable and sustainable for the individuals and their family members ^30^. 

### Strengths and limitations

The study sample was representative of the population of Kollam District in Kerala. To the best of our knowledge, the impact of self-care activities on BP control is not studied in detail in community settings in India. Due to the self-reported nature of adherence pattern, the study is prone to recall and response bias. The associations observed in our study may not infer causality due to the cross-sectional nature of the study.

## Conclusion

Overall, BP control is achieved in two of five participants with diagnosed hypertension and on treatment in Kerala at the community level. Further, compliance to self-care practices are less than optimal in the study population. Optimal adherence to self-care strategies is important in improving BP control rate in individual with hypertension. Family support is key to improving adherence to self-care practices and thereby facilitates individuals with hypertension to achieve improved BP control. Interventions to improve family support for self-care activities could have a significant public health impact in achieving better population-level BP control rates in Kerala, India. 

## Data availability

### Underlying data

Figshare: Role of family support and self-care practices in blood pressure control in individuals with hypertension; results from a cross sectional study in Kollam District, Kerala. https://doi.org/10.6084/m9.figshare.12616325.v1 ^18^.

This project contains the following underlying data:

- Dataset.csv (raw individual level data for each participant)
- Codes used in dataset.docx

### Extended data

Figshare: Role of family support and self-care practices in blood pressure control in individuals with hypertension; results from a cross sectional study in Kollam District, Kerala. https://doi.org/10.6084/m9.figshare.12616214.v4 ^16^.

This project contains the following extended data:

- Interview schedule English version.pdf

Figshare: Role of family support and self-care practices in blood pressure control in individuals with hypertension; results from a cross sectional study in Kollam District, Kerala. https://doi.org/10.6084/m9.figshare.12662042.v2 ^17^.

This project contains the following extended data:

- Interview schedule malayalam version.pdf

Figshare: Role of family support and self-care practices in blood pressure control in individuals with hypertension; results from a cross sectional study in Kollam District, Kerala. https://doi.org/10.6084/m9.figshare.12662357.v1 ^13^.

This project contains the following extended data:

- Participant information sheet english version.pdf
- Participant information sheet malayalam version 2.pdf

Data are available under the terms of the Creative Commons Attribution 4.0 International license (CC-BY 4.0).
