# Supplementary material for: Role of family support and self-care practices in blood pressure control in individuals with hypertension: results from a cross-sectional study in Kollam District, Kerala
Source: Wellcome Open Res. 2020 Jul 28;5:180. [Version 1] doi: 10.12688/wellcomeopenres.16146.1 (PMC7713892; doi:10.12688/wellcomeopenres.16146.1)
Supplement: Peer review history

# Peer review: Reviewer response for version 1

**DOI:** 10.21956/wellcomeopenres.17726.r41518

**Published:** 2020-12-02

**Authors:** Timothy Walker

**Affiliations:** 1 School of Medicine and Public Health, University of Newcastle, Newcastle, Australia

This is a well conducted and clearly described cross-sectional study assessing blood pressure control practices among a cluster-derived sampling of adult hypertensive patients in Kerala, India, and correlating this with blood pressure control at a single time point.

 The sampling methodology is very clearly described. 100% participation is remarkable, and may reflect additional cultural or other factors at play.

 The H-SCALE tool used was put through a rigorous translation, piloting and validation process prior to its use in the study. Results are clearly presented and well laid out. Generally it is clear what was found, and the authors present a sensible discussion of their results in light of the available literature.

 A few minor suggestions for improvement follow below:

1. The p-values in Table 1 are not clearly described or annotated to make clear which statistical test was applied, or what question was to be answered by this test. It is generally preferable if a table can stand alone without further explanation, and an explanatory note should be added to Table 1 to aid interpretation.
2. The statistical methods used could be laid out more clearly. It is unclear which tests were used to arrive at the stated p-values in the Tables, and no check for normal distribution of data appears to have been undertaken prior to use of parametric tests. Overall the statistics are inadequately described to allow replication of results.
3. Using a single set of three blood pressure measurements to define a concept as complex as blood pressure control is obviously methodologically fraught. While this may well have been the most practical method available, this limitation should have been discussed, particularly given it related to the primary outcome.

Thanks for the opportunity to review this interesting study.

Is the work clearly and accurately presented and does it cite the current literature?

Yes

If applicable, is the statistical analysis and its interpretation appropriate?

Yes

Are all the source data underlying the results available to ensure full reproducibility?

Yes

Is the study design appropriate and is the work technically sound?

Yes

Are the conclusions drawn adequately supported by the results?

Yes

Are sufficient details of methods and analysis provided to allow replication by others?

Yes

Reviewer Expertise:

Gastroenterology, Internal Medicine, Global Health, Medical Education.

I confirm that I have read this submission and believe that I have an appropriate level of expertise to confirm that it is of an acceptable scientific standard.

# Peer review: Reviewer response for version 1

**DOI:** 10.21956/wellcomeopenres.17726.r41087

**Published:** 2020-11-13

**Authors:** Yogesh Kalkonde

**Affiliations:** 1 Society for Education, Action and Research in Community Health, Gadchiroli, India

In this manuscript the authors evaluated the role of family support and self-care on blood pressure control in Kollam district of India. The study found that blood pressure control was poor (40%) and certain self-care activities and good family support were associated with improved blood pressure control. These findings support evaluation of self-care and family-support interventions to improve control of hypertension which is a leading risk factor for death and disability globally. There is very little information on self-care and family support for hypertension control in community settings in India so this study is important.

 It is a well conducted study. The authors could get a response rate of 100% which is really hard to achieve in field studies.

 I have some suggestions which are listed below.

 Title

- The title needs to reflect that the study was conducted in India. Abstract

The abstract is concise.

- The authors should define BP control in the abstract by specifying the cut offs e.g. <140/90.

Introduction

 The introduction is well written, explains the rationale for conducting this study and provides the objectives of the study. 

- The statement ‘disproportionately higher number of deaths from elevated BP in LMICs…attributable to suboptimal control of BP’ needs a reference.
- The statement ‘self-care has been recognized as an important determinant…at the individual level’ also needs a reference.

Methods

 The methods section is clearly written.

- It would be beneficial for the readers if the authors can provide the rationale for conducting this study in the Kollam district of Kerala.
- The authors state that they drew 23 individuals with hypertension randomly from the population-based listing of all individuals with diagnosed hypertension. However, the source of this list is unclear. Was this list prepared during the population-based screening of hypertension under the national programme for prevention and control of chronic diseases? Was the list obtained from the primary health centres?
- The authors state that self-care activities were captured using H-SCALE tool. The authors have attached the tool in the supplement but it will be beneficial for the readers to describe in brief what the key components of this tool are in the methods section.
- In defining medications adherence, adherence to DASH diet, physical activity engagement, weight management, family support the authors provide certain cut-offs to classify these activities as good or mild, moderate and strong. What was the rationale for selecting these thresholds? It will be good to state this in the methods.

Results

- In Table 2, the authors should provide data on percentage of men and women with controlled BP in addition to providing mean systolic and diastolic BP.
- In Table 3, the categories in the duration of hypertension are listed as <5 years, 5-10 years and <10 years. The last category should be >10 years.
- The authors may want to change the title of Table 4 to ‘strength of association of components of self-care and control of blood pressure’.
- In Tables 3 and 4, the terms non-smoking adherence and alcohol abstinence should be replaced with simpler terms such as current smoking and current alcohol use. Understanding non-smoking non-adherence can become challenging.
- The authors use weight management adherence as one of the parameters to assess self-care but have not provided an information on obesity among the participants. It will be useful to provide this information.

Discussion

 The authors have discussed the interpretation of their findings, the strengths and the limitations of their study. However, some additional discussion will be useful.

- Physical activity and weight management are important measures to improve blood pressure control. However, these factors were not associated with blood pressure control in this study. The authors may want to discuss briefly why this might be the case.
- While discussing no association between alcohol use and BP control the authors argue that this could be related to the amount of alcohol consumed but a more plausible reason in this study could be that a very few participants were using alcohol - 86.7% men and 100% women participants were alcohol abstinent. Therefore, finding an association between blood pressure control and alcohol use could be hard.
- Presence of other diseases e.g. diabetes, coronary artery disease and stroke among patients with hypertension might modify self-care and control. As the data on these comorbidities are not presented, this should be discussed as a limitation of the study.

Overall it is an excellent study which meets the standard of the journal and will advance this area of research further in India.

Is the work clearly and accurately presented and does it cite the current literature?

Yes

If applicable, is the statistical analysis and its interpretation appropriate?

Yes

Are all the source data underlying the results available to ensure full reproducibility?

Yes

Is the study design appropriate and is the work technically sound?

Yes

Are the conclusions drawn adequately supported by the results?

Yes

Are sufficient details of methods and analysis provided to allow replication by others?

Yes

Reviewer Expertise:

Public health, non-communicable diseases, epidemiology.

I confirm that I have read this submission and believe that I have an appropriate level of expertise to confirm that it is of an acceptable scientific standard.
